# An iPad-Based Tool for Improving the Skills of Children with Attention Deficit Disorder

**DOI:** 10.3390/ijerph120606261

**Published:** 2015-06-02

**Authors:** Natalia Wrońska, Begonya Garcia-Zapirain, Amaia Mendez-Zorrilla

**Affiliations:** 1 International Faculty of Engineering, Lodz University of Technology, Żwirki 36, Łódź 90-924, Poland; E-Mail: nwronska@deusto.es; 2 The Deustotech-LIFE (eVIDA) Research Group, Faculty of Engineering, University of Deusto, Bilbao 48007, Spain; E-Mail: amaia.mendez@deusto.es

**Keywords:** attention deficit disorder, serious games, comprehension, iPad

## Abstract

Attention Deficit Hyperactivity Disorder (ADHD), with a worldwide prevalence of 5.29%–7.1%, is one of the most common neurodevelopmental disorders among children and adolescents. Apart from typical symptoms like inattention, hyperactivity and impulsiveness, patients also evidence attention deficit problems with reading comprehension. This in turn causes poor school performance and widens the gap with peers without ADHD. This paper presents a novel and interactive tool based on Serious Games for Health, whose aim is not only to improve comprehension, but also hold the user’s attention. This tool is geared towards assessing reading quality and is intended for iPad devices. Preliminary results obtained from the experiment performed to evaluate the game are included in this report. A group of six typically developing children from Colegio Vizcaya aged between 8 and 12 took part in the evaluation of motivation, satisfaction and usability of the same therapy in the new media. Results obtained by participants playing the game were analysed together with questionnaires concerning the usability of the system. Game evaluation resulted in relatively good statistics-average score was 3 points out of 4 and average time for completing the exercise was 59 seconds. A SUS questionnaire with an average score of 92.75 out of 100 indicates that the game presented is user-friendly and an effective tool. Moreover, based on the feedback obtained from participants, the game had been improved and additional functionality introduced. Older participants completed the first game faster than the younger ones, but age was not influential in subsequent games.

## 1. Introduction

Attention Deficit Hyperactivity Disorder (ADHD) is a substantially common diagnosed neuro-behavioural disorder in children and adolescents (<18 years). Worldwide prevalence is between 5.29% and 7.1% [1]. Symptoms of ADHD differ depending on the case. Some children can suffer from inattention only, others from hyperactivity or impulsiveness, and there is also a group which evidences all the symptoms [2]. The inattentive type can be distracted easily, has problems with focusing their attention and gets bored fast. On the other hand, hyperactivity is characterised by an inability to sit still and perform quiet tasks, talking nonstop and being permanently in motion. Impulsivity includes symptoms such as impatience, acting without being concerned about the consequences and interrupting conversations [3].

For diagnosis to be confirmed, the symptoms of ADHD should be noticeable at ages 6–12 and maintained for more than six months [4]. There are two main criteria for diagnosing ADHD—DSM IV [5]. Usually used in the USA, and ICD-10 [6]—more common on the European continent. The second criterion is more restrictive and therefore is therefore less easy to identify than the first one [7].

There are different treatment methods available, depending on age of the child. The first step in the case of pre-school children is a parent training/educational programme [7] because drug treatment is not recommended due to unknown long-term effects [8]. School-age children in first-line treatment require group/individual psychological treatment. Drug treatment is advised for children with serious symptoms and impairments or for those with moderate symptoms if they have refused non-drug interventions. In addition, children who provide an insufficient response to parent training/educational programmes or group psychological treatment should undergo pharmacological treatment. There are several drugs available for such treatment, which have different applications. However, all of them cause side-effects to a greater or lesser extent [9].

Despite the symptoms stated above, children with ADHD also often demonstrate difficulties in reading comprehension [10]. Although they read fluently, understanding what they read is a problem related to one of the executive functions, which is working memory. Distraction usually occurs while reading longer texts because of difficulty in monitoring what was read [11]. Reading fluency is linked to a fundamental cognitive process-processing speed-which is required to complete the task with reasonable accuracy [12]. The lack of reading fluency resulting in problems with comprehension impairs working memory, because word decoding requires a higher-level process for the same time-limited resources [13]. Therefore, an improvement in working memory is closely related to an ability of children with ADHD not to be distracted so fast. Working memory training contributes to positive effects by either increasing attentiveness or decreasing hyperactivity, and therefore improving school performance and attention when reading and observing pictures [14]. Working memory training sessions are usually conducted on computers via the possibility of adjusting feedback as a form of reinforcement [15]. Furthermore, results obtained from research conducted by Prins *et al.* [16] show that performing working memory training on a computer when applying game conditions significantly influence children’s motivation and performance.

To minimise ADHD symptoms, children must be motivated and stimulated effectively, and this is possible by using computers and games in therapy [17]. In his paper [18], Kulman explains why using video games and digital media helps children with ADHD in therapy. He claims that playing a video game involves the use of executive functioning skills and also organisational and metacognitive skills. The most crucial skills, which are worked on while playing games, are memory skills and attentional skills. Due to the fact that kids with ADHD get bored fast, it is important to constantly catch their attention. Video games enable such conditions because they require that a child stays focused and engaged the whole time, and also that they engage all the senses, which makes them more interesting for the user. Another problem linked to attention deficit is lack of “immediate reinforcement or consequence” [18]. In cases where children are doing their homework and do not get any signal or response that it is being done correctly (or not), they get distracted and lose attention. Video games ensure a sense of progress because they provide the user with feedback, such as by scoring or collecting some items. All the factors described above encourage us to adopt video games and digital media in therapy of children with ADHD.

Taking into account the problem with attention and comprehension while reading and looking at pictures and the positive influence of games on children with ADHD, the idea came about to improve attention skills by playing the game. Therefore, the aim of this report is to present a new interactive tool which supports children with ADHD. This tool was tested in El Colegio Vizcaya who based their therapies on materials available at a referenced Spanish educational site called “Orientation Andujar” [19]. The tool uses a game-based learning approach to improve reading comprehension skills in children. By converting paper-based exercises used thus far in ADHD therapy, the author created the game (called “LyC: Lectura y Comprensión”; English translation: Reading and Comprehension) developed for iPad, which is based on techniques used in Serious Games for Health. It is foreseen that this game will have a positive impact on progress in attention and comprehension while children with ADHD read.

The remainder of this paper is organized as follows: Section 2 presents a review of the literature concerning games created for children with learning difficulties and special needs. Materials and methods used in development of the game will then be described, while Section 4 contains a description of the system’s design. The last section includes results and conclusions.

## 2. Background

This section presents a review of the literature concerning the previous work. It includes a description of the influence of computer game-based learning and Serious Games on education, particularly special education. Below is provided information about applications and games created for children with learning difficulties and disabilities.

There are plenty of Serious Games created for children with special needs and devices for which these games are developed are also numerous, starting with PCs, along with smartphones and tablets and ending with consoles and biofeedback. In the particular case of games created for children with Attention Deficit Disorder, they mainly focus on enhancement of attention by performing some general cognitive exercises. None of them has been directly created for the purpose of improving attention and comprehension skills.

Furthermore, iPads and iOS devices in general are rarely incorporated in games for the special education sector, especially for children with ADHD. Compared to applications and games created for the Android mobile operating system, iOS is a less common choice for developers of Serious Games. Considering the fact that Apple’s mobile devices are becoming more and more popular on the market, this gap should be filled in order to ensure access to educational help for iOS users.

To conclude, it can be stated that the game presented is a pioneer in its field, not only because it is intended for a niche device, namely the iPad, but also because it provides an educational tool for children with Attention Deficit Disorder.

There are several examples of different tools used to help children in developments we have found in different countries, although general tools are taken into consideration in the cases shown in Table 1 from Canada, Egypt and the USA for children with special needs or disabilities. The type of technology used—whether PC or Smart Devices—is not a critical issue when distinguishing between applications—what really makes then differ is that in the cases shown from Malaysia, Spain and the USA (the first three cases shown in Table 1), tools are offered that deal with a specific problem that has been diagnosed in children, such as dyslexia or autism. No special relevance is placed on the inclusion of biofeedback techniques for objective evaluation purposes in these developments.

Table 2 shows examples of technological solutions where the application varies from PCs to smartphones to Wii consoles. Each of these types of software is specifically geared towards dealing with the most common problems found in children with ADHD: fostering attention, time management, and lack of social skills. However, we have included an example of a general technical nature in the the table which is common to all types of group, such as relaxation and breathing. Attention should be drawn to the fact that when the application allows it–which often occurs in solutions for PCs-there is a tendency to include biofeedback techniques to more objectively assess the impact of therapy on such individuals.

The need can be seen in both Table 1 and Table 2 to steadily move towards solutions that include Cloud Computing or Cloud Analysis, so that both users and therapists who are supervising them may gain straightforward access to results and to a historical analysis of evolution.

Table 3 shows some examples of usability testing of software systems and web platforms from various research projects from 1987 to the present. The authors of the paper along with the school team involved in the investigation decided to use SUS. Due to the extensive use of SUS by the scientific community, this enables easier comparison of the usability of our tool against others.

**Table 1:** Examples of applications geared towards children with special needs.

Author	Game Name	Year	Type	Country	Objective	Technologies
Rozita Ismail, *et al.* [20]	−	2011	Specific	Malaysia	Teaching dyslexic children Malay language.	PC
Zelai Saenz de Urtuti, *et al.* [21]	−	2011	Specific	Spain	Teaching First Aid to children with ASD.	Mobile devices: Android
Juan Pablo Hourcade, *et al.* [22]	−	2012	Specific	USA	Enhancing social skills of children with ASD.	Mobile devices: Android
Moutaz S. Saleh, *et al.* [23]	−	2012	General	Qatar, Canada	Improving the knowledge of children with special needs.	PC, TUI
Ghada A. El Khayat, *et al.* [24]	IWASGS (Intelligent Web-based Adaptive Serious Games System)	2012	General	Egypt, USA	Improving the knowledge of children with learning disabilities.	PC
Rachelle Campigotto, *et al.* [25]	MyVoice	2013	General	Canada	Supporting people with memory and communication deficit.	Mobile devices
Supawade Cindy Lee [26]	−	2013	General	USA	Improving children’s handwriting and visual perceptual skills.	Mobile devices: Android: iOS

**Table 2:** Specific applications for children with ADHD.

Author	Game name	Year	Type	Country	Objective	Technologies	Online?
B. H. Cho, *et al.* [27]	Attention Enhancement System	2002	Specific	Korea	Fostering attention of children with ADHD.	PC, Biofeedback (EEG), Virtual Reality	NO
Krestina L. Amon, *et al.* [28]	The Journey to Wild Devine	2008	General	Australia	Teaching breathing and relaxation techniques of children with ADHD.	PC, Biofeedback (heart rate and skin conductivity sensors)	NO
N. Aresti Bartolome, *et al.* [29]	−	2010	Specific	Spain	Improving the social skills of children with ADHD.	Wii console	NO
Tsung-Yen Chuang, *et al.* [30]	Wii play, Wii sport	2010	Specific	Taiwan	Fostering attention of children with ADHD.	Wii console	NO
Lim CG, *et al.* [31]	CogoLand	2012	Specific	Singapore	Fostering attention of children with ADHD.	PC, Biofeedback (EEG)	NO
Maite Frutos- Pascual, *et al.* [32]	−	2013	Specific	Spain	Improving time management skills and task prioritisation of children with ADHD.	Web-based multimedia application	YES
Ruiz-Manrique *et al.* [33]	ADHD Trainer	2014	Specific	Spain	Cognitive skills improvement of children with ADHD	Mobile application	YES

**Table 3:** Usability questionnaires examples.

Author	Acronym	Year	Institution
Kirakowski J. [34]	SUMI	1987	
Davis, F.D. [35,36]	TAM	1989	
Brooke, J [37]	SUS	1996	
Lin, Han X. *et al.* [38]	PUTQ	1997	Purdue
Lund A. M. [39]	USE	2001	Sapient
Geneva Emotion Research Group [40]	GAQ	2002	University of Geneva
Donker, A. [41]	Fun	2005	Vrije Universiteit

## 3. Materials and Methods

This section describes the methods and materials applied in development and evaluation of the game for the iPad. The game is created for children with ADHD and its purpose is for them to gain a command of reading comprehension by performing specific exercises.

### 3.1. Participants’ Description

The application was evaluated by the control group of six typically developing children (two boys and four girls), aged between 8 and 12 years old, who have not been diagnosed with ADHD, from Colegio Vizcaya in Bilbao, Basque Country, Spain. For ethical reasons, a consent form signed by the parents of participants was required. The study was conducted in accordance with the Declaration of Helsinki, and the protocol was approved by the Ethics Committee of the University of Deusto (financed by Project P6750).

### 3.2. Materials

Development of the game was based on the exercises fostering attention during reading comprehension and pictures observation, which are available on the website [19]. These exercises need to be printed and solved using a pencil. The main purpose of this project was to convert paper-based exercises into a game-based version available for iPad devices.

The application consists of nine interactive exercises with different difficulty levels defined by psycho-pedagogy experts from [42]. The game process includes three stages. Firstly, the user has to read the text, which contains the important information. Then, the information must be understood and processed into the answer. The last step is to select the correct answer from all those presented. The answer is selected by dragging the button from the toolbox and dropping it on the picture or label. Drop action causes the change of picture or label according to the button, which was chosen from the toolbox. The following tools were employed in developing the game:

- iPad 2-Apple’s multi-touch portable device based on iOS 7.
- XCode 5-programming platform based on objective C# programming language and Interface Builder for visualising the view.
- SQLite database used initially for collecting the user’s results locally on the device, to ensure that therapists would gain access to them only from the iPad used.
- MySQL database created for sending the results to the server used in an improved version of the game, as the previous database limited the application’s mobility.

### 3.3. Methodology

The trial was conducted with use of an iPad that had been installed with the game being presented. Children were asked to perform the tasks carefully and without hurry. During each trial points gained by the user and time needed to complete the task were monitored. The following step was to respond to usability questionnaires rating the game, its difficulty and visual aspects.

The whole process involving creation of the game took place in several phases.

**Figure 1:**
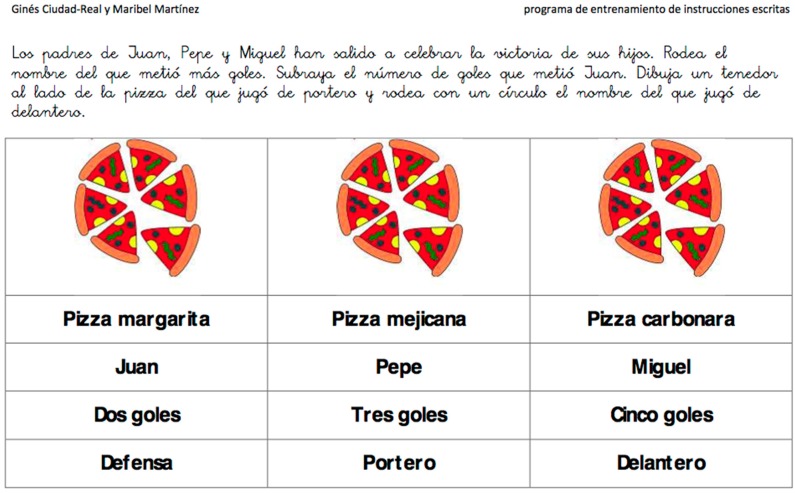
Example exercise used thus far in therapy [19].

**Figure 2:**
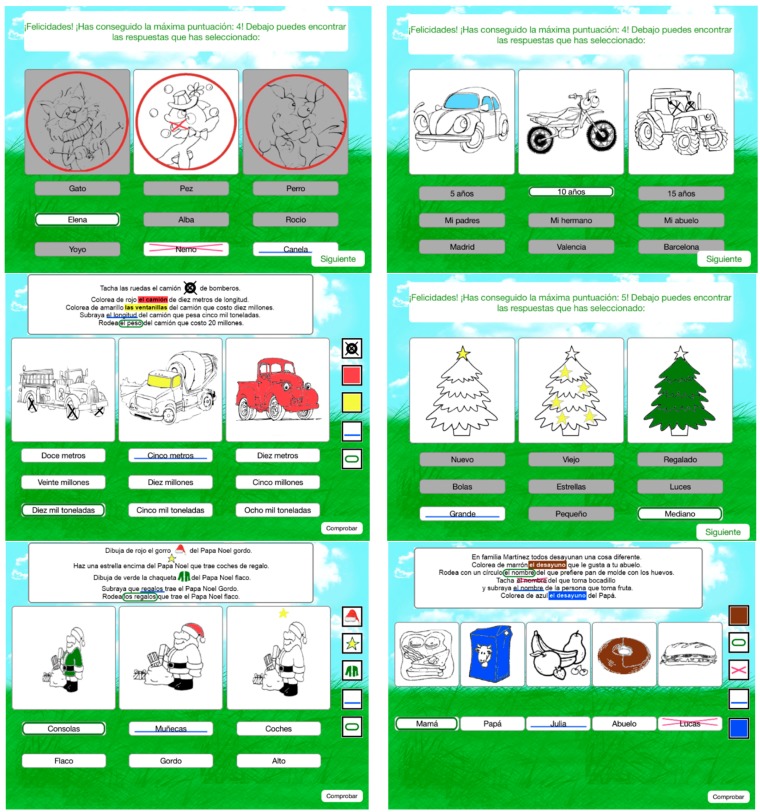
Screenshots of the application.

In the first phase decisions concerning the technical details were made. From the many devices available on the market, the iPad was chosen because of being popular and widely used in the education sector. In addition, owing to its mobility, interactive multi-touch display and entertaining nature, it is more attractive to the potential user. Next, exercises from the list available on the website [19] were selected based on the possibility to adapt them to the game scenario. The example exercise is shown in Figure 1 to visualise the resources used thus far by therapists. The same exercise converted into an iPad version will be shown in Figure 2.

The following phase included the development process of the beta version of the game and verification by an expert-in this case, Patricia Clemente, Head of the Psychology Department at Colegio Vizcaya. Taking into account all the suggestions made by the expert, the game was improved and prepared for evaluation with children from Colegio Vizcaya.

The next phase-evaluation-is very important in developing the software because based on the results and opinion of the control user the game can be analysed and adapted appropriately for the end-user-the ADHD child. The game was tested in a class environment at Colegio Vizcaya, with each participant testing the application and filling out the questionnaire individually by themselves (though all had the support of a teacher if they needed help). At the beginning of the game, it displayed instructions on how to play and an explanation of the functions used, e.g., types of button, drag and drop function, *etc*. Due to the fact that children participated in evaluation during class hours, there was not enough time for each of them to do all the exercises. Thus, participants only did the first three exercises. The Spanish version of the SUS (System Usability Scale) [27] was used in the case of questionnaires. The questionnaire consisted of ten items, each of which was to be assigned using the Likert scale ranging from 1 “strongly disagree” to 5 “strongly agree”. The game was improved by adding additional functionality based on participants’ opinion. The last phase involved applying the MySQL database to store the results on the server.

## 4. System Design

**Figure 3:**
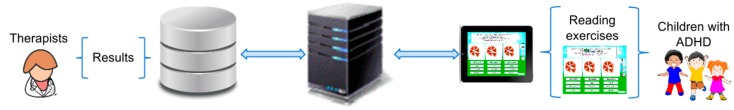
General outline of interaction in the system.

The system’s design is described in this section, including all the processes which take place during its operation. A general outline of the system’s operation is provided to visualise the process. As can be observed in Figure 3 when the user (in this case the child with ADHD) interacts with the system by playing the game, the data that is introduced is then retrieved by the system. The data is then passed on to the database to be stored on the server. Access to the results is provided for the therapist to enable progress to be monitored.

### 4.1. High-Level Design

**Figure 4:**
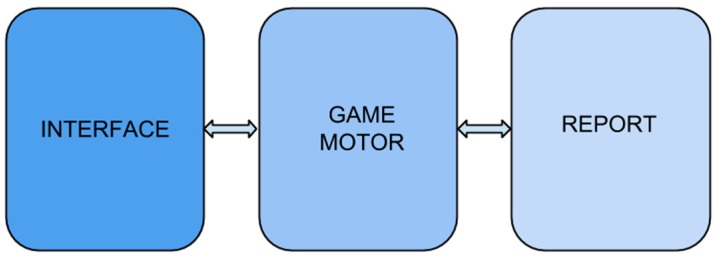
High-level design diagram.

A general description of the entire system is provided in this subsection with the elements that make up the game being identified. Figure 4 shows a diagram of high-level design. As can be observed, the architecture used in designing the game consists of three main elements: interface, game motor and report. The design is segmented and the elements separated according to their functions. Each element is described briefly below.

The *interface,* also called *presentation layer,* provides interaction for the user’s output/input. In the case of the game presented this function is performed by the iPad’s multi-touch screen, which displays exercises (output) and records touches by the user (input). The interface is connected to the next element-the game motor-which is responsible for sending the code to be used and touch recognition. Once the touch is recognised and a score assigned, it is then sent to the interface from the game motor and displayed to the user after completing each exercise.

The *game motor,* also called *business or logic layer*, is responsible for the functioning of the game. It is connected both to the interface, sending the information to be displayed on the screen and recognising the touch events performed while the game is being played. The game motor is also connected to the reporting part by sending the game’s parameters (user name, score, time needed to complete the exercise, time needed to check the results and number of repetitions) to be stored. This element was created and managed using XCode 5 development software.

The *report,* also called *database layer,* manages all the processes for storing the results obtained in the game, and which are prescribed to the user name. It is connected to the game motor as part of the process involving retrieving the data. These results are then displayed in the form of a table to the therapist and are accessible from any device with Internet connection. Database access protocol was created in the source code in order to manage this element.

### 4.2. Low-Level Design

In this subsection A detailed description of the three main elements making up the system are described in this subsection–interface, game motor and report-together with the diagram showing the operation.

The interface is responsible for interaction with the user. In case of the game presented, the interaction takes place using the iPad’s multi-touch screen, which plays a double role. Firstly, it provides the output for the user by displaying the game content and secondly, it provides the user’s input by recording all touch events. The interface element also includes the way that the device communicates with the user, and so the flowcharts describing the game content are shown in this part.

**Figure 5:**
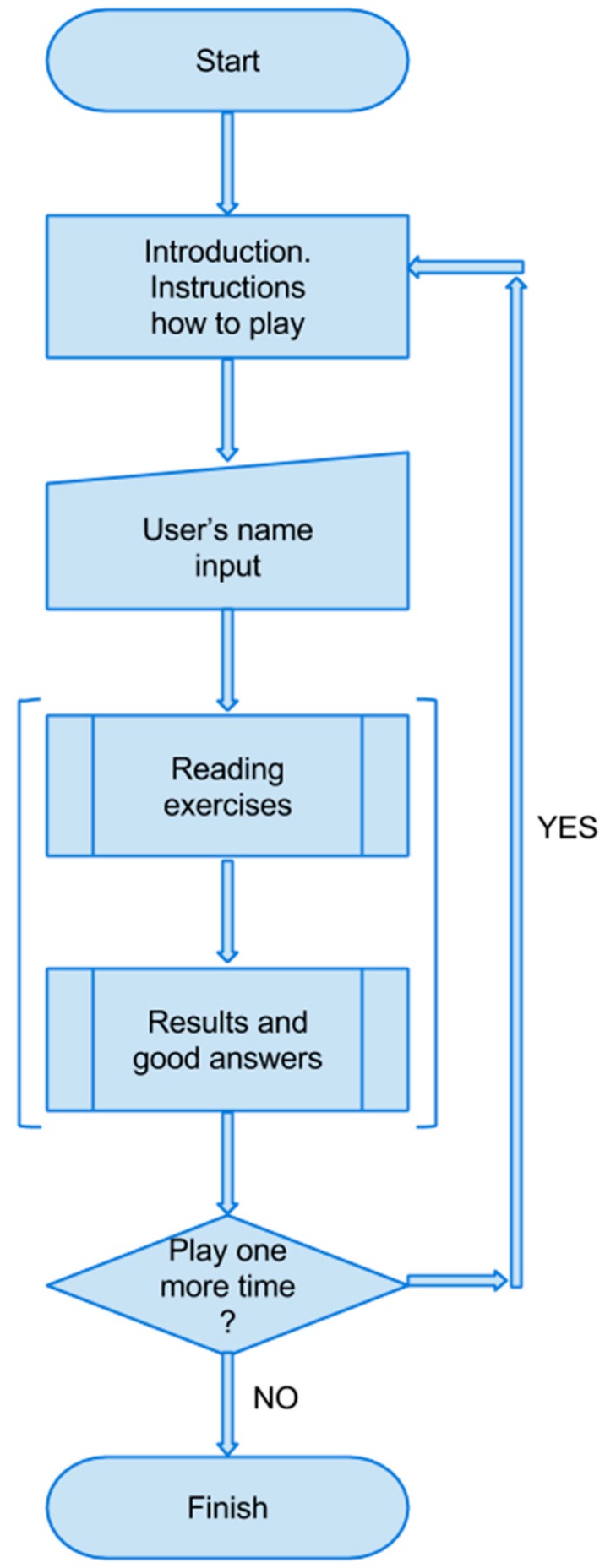
General flowchart of the game.

Figure 5 shows the general flowchart of the game presented. As can be observed, the introduction with instructions on how to play is displayed at the beginning. The next view is the interactive window that asks the user to input his/her name. The following step is a predefined process of how to play the game, which is described in detail further in this section. After finishing each game, the user is then provided with the results with correct answers. Due to the fact that the game presented consists of 9 exercises, the previous two steps are repeated nine times. After the last exercises, the window which gives the possibility to play again is displayed. If this option is chosen, then the user can play again. Otherwise, the game is finished.

**Figure 6:**
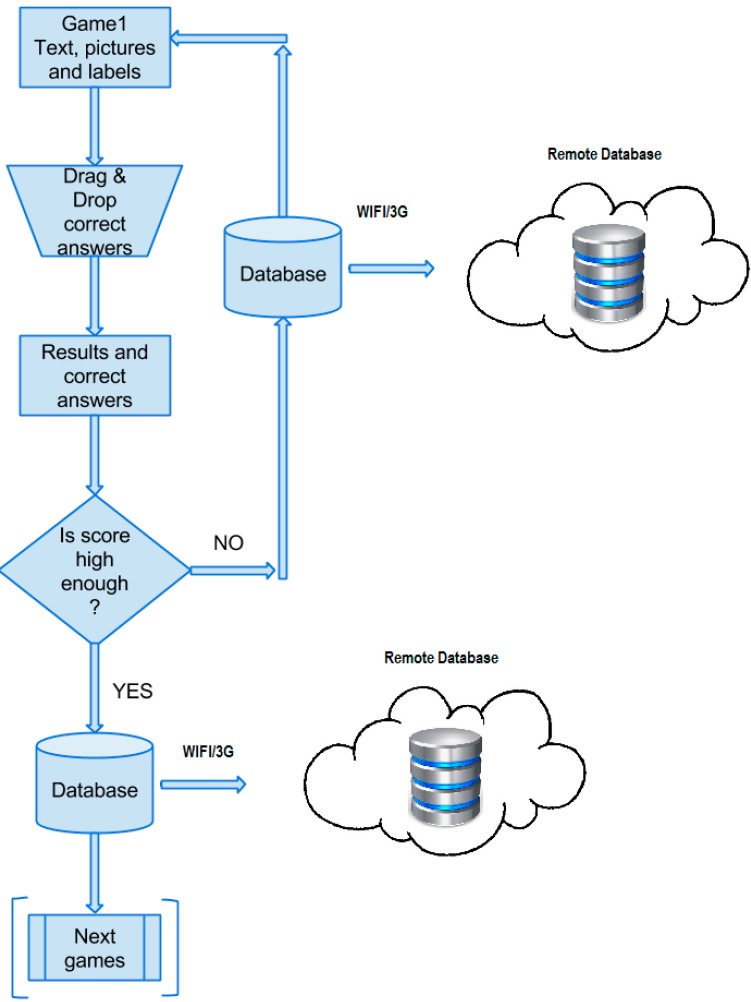
Process diagram of the game.

Figure 6 contains a detailed description of the predefined process included in the general diagram of low-level design-the flowchart of the game. Due to the fact that all nine exercises have the same operation principle, only one game is shown, which then can be replicated. The window with the following elements appears just after starting the game: text (on top of the view), pictures (below the text), labels (below the pictures) and draggable buttons (on the right side of the view). Depending on the game, different numbers of the above elements are displayed. At this stage, the user’s task is to read the text carefully and decide about the correct solutions. Next, using draggable buttons, the user can drag the button itself and drop it on the picture or label. Drop action causes the change in picture or label in accordance with the feature prescribed for the button. After completing all tasks, the next view with results and correct answers is displayed. Only if the number of points is equal to the maximum or (maximum-1) can the user proceed to the next exercise. Otherwise, the game must be repeated until the score is high enough to pass to the next exercise. Both moving on to the next exercise and playing again initiates the process involving sending the results to the database. The last block in the flowchart stands for the predefined processes of the remaining eight games.

The game motor is responsible for how the game functions. It is connected both to the interface, by sending the information to be displayed on the screen, and to the reporting part, by storing and accessing the data. This connection is provided and managed by an XCode 5 integrated development environment. XCode combines both objective C programming language for implementation of source code and Interface Builder for the graphic design in order to create the functioning user interface for Apple devices. Once the game is displayed, the timer starts to record the time of the user’s interaction. Touch action is analysed and once the game is finished, the timer stops. All the parameters collected by the user while playing the game are then prepared in order to be sent to the database. The score obtained is displayed to the user in the interface and at this stage, operation of the game motor ends.

The reporting element of the game presented is responsible for storing the results obtained while playing the game in the database. These results are prescribed to the user name, which was introduced at the beginning of the game. The connection with the game motor is established via a code in XCode so that processes involving storing and retrieving the data can be maintained. The local database was implemented using SQLite and the all the results were always stored on the device. Thanks to collaboration with another project developed at DeustoTech it was possible also to store the dataon a remote server. To do so, MySQL database was adapted, enabling the data to be accessed from any device through to the Internet (when they have connectivity). The game parameters in accessible format for the database are received in this part. The URL request is then created to allow connection with server. Once the connection is established using 3G/4G or WIFI, the results obtained by the user together with his/her name are then sent to the database. After this, a response from the server is obtained. The results sent to the database are assigned to variables created in the server and the table is then initialised both with variables and values. The table is displayed in the game manager so that the therapist may gain access to it.

## 5. Results

The experiment conducted on the control group is described in this section. The results were obtained on 27 May 2014 at Colegio Vizcaya and include evaluation of two tests. The first one is a tool evaluation, which contains the results of children playing the game. The second one is an evaluation of the system’s usability based on the SUS questionnaire. Six children (two males—33% of the participants—and four females—67%) aged between eight and twelve years old took part in the evaluation. 

First of all, participants read the instructions carefully and introduced their codes in the name text field (to preserve anonymity, participants used a code instead of their real name). They then explored the tool by playing the game individually. Students had to follow the three stages: reading the text, which contains the important information; understanding and processing information into answers; selecting the correct answer from all presented by dragging the button from the toolbox and dropping it on the picture or label.

Two parameters were recorded during evaluation of each child-time needed to complete each exercise and obtain a score (if students do not respond correctly to any examples in any exercise they will score 0). These results were sent to the SQLite database, which stored them locally on the device. Access to this data was provided only by the iPad used.

Just after finishing playing the game, children were asked to fill out the SUS questionnaire. The usability of the game was evaluated with the Spanish version of the SUS questionnaire. The questionnaire consisted of ten items, each of which was to be assigned using the Likert scale, ranging from 1 “strongly disagree” to 5 “strongly agree”.

The lists of questions asked in the SUS questionnaire are provided below:

1. I think that I would like to use this system frequently.
2. I found the system unnecessarily complex.
3. I thought the system was easy to use.
4. I think that I would need the support of a technical person to be able to use this system.
5. I found the various functions in this system were well integrated.
6. I thought there was too much inconsistency in this system.
7. I would imagine that most people would learn to use this system very quickly.
8. I found the system very cumbersome to use.
9. I felt very confident using the system.
10. I needed to learn a lot of things before I could get going with this system.

The following subsections contain a detailed analysis of the results obtained during evaluation of the tool and its usability.

### 5.1. Game Evaluation

**Figure 7:**
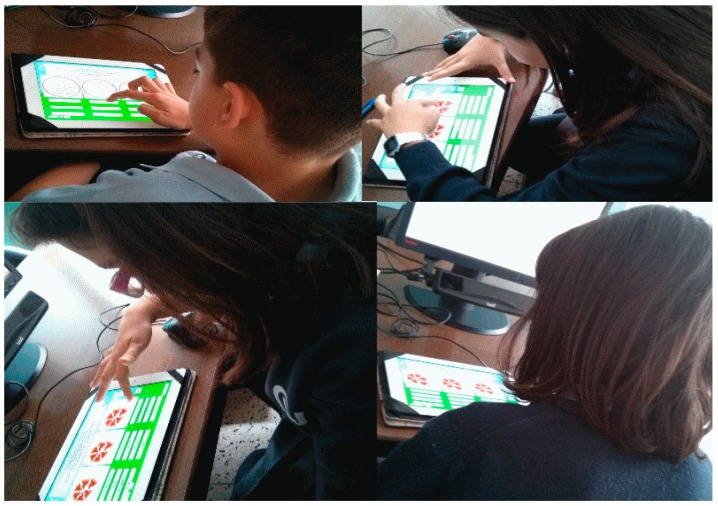
Children evaluating the game.

The game evaluation is described in this section along with a statistical analysis of preliminary results. Children playing the game are shown in Figure 7. To preserve anonymity, photos were taken from the side, not showing participants’ faces.

The results obtained by participants are shown in Table 4. The table contains the time taken and score obtained by each participant in each game and also statistical calculations of the mean value of time, score and their standard deviation. To preserve anonymity, participants are identified by their code rather than by name.

**Table 4:** Results obtained by participants in evaluation of the game.

User ID		Game 1	Game 2	Game 3
ai123b	Time	99	41	40
	Score	3	4	4
in143b	Time	99	78	31
	Score	3	0	3
co156b	Time	72	31	31
	Score	4	4	4
lo163b	Time	112	67	70
	Score	4	4	0
jp176b	Time	70	43	33
	Score	0	3	4
iu216b	Time	65	42	35
	Score	3	3	4
**Statistics**		**Game 1**	**Game 2**	**Game 3**
Mean time		86.17	50.33	40.00
Standard Deviation		19.53	18.04	15.07
Mean score		2.83	3.00	3.17
Standard Deviation		1.47	1.55	1.60

**Figure 8:**
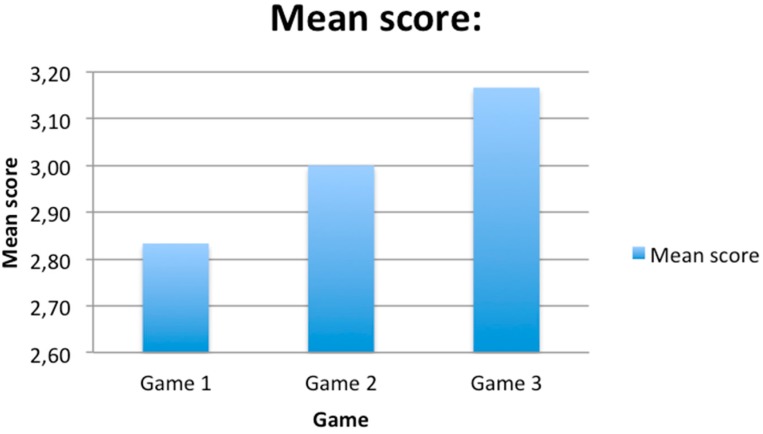
Graph representing the mean score obtained in each game.

**Figure 9:**
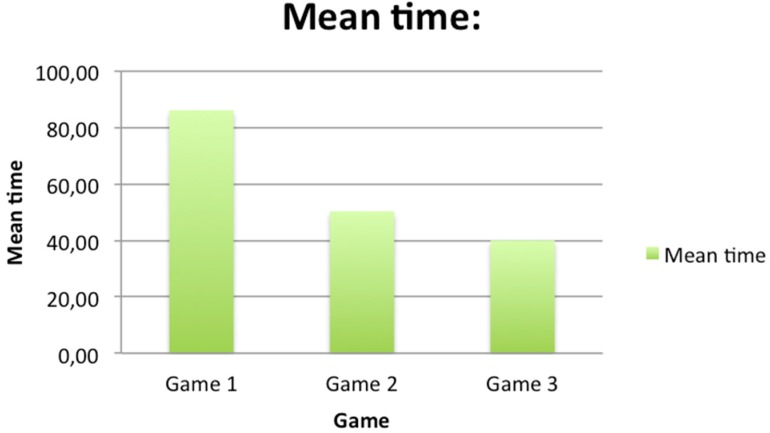
Graph representing the mean time obtained in each game.

**Figure 10:**
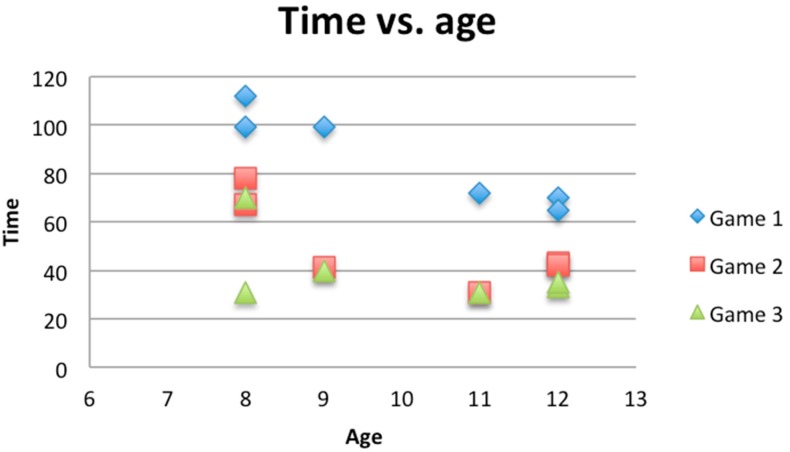
Graph representing the time with respect to age of participants.

In this sample, it can be observed that in the first game the time needed to complete the exercise is generally longer than in the remaining two games. Comparing the two graphs (Figure 8 and Figure 9), it can be seen how the mean time changed with respect to mean score. The time needed to complete the game shows a decreasing tendency, whereas the score obtained shows an increasing tendency. However, in the following games, the time parameter improved, as participants had already learnt how to play. As regards standard deviations, it can be observed that they decrease, which means that divergence of results also decreases. Furthermore, the time needed to complete each game will be compared to the age of each participant (see Figure 10).

### 5.2. System Usability Evaluation

The user-experience evaluation is described in this section, and the results obtained from the SUS questionnaire are provided in Table 5. During usability evaluation, all the children had a teacher who helped them assess the tool.

In the case of odd-numbered questions the best score is 5, whereas in the case of even-numbered questions the best score is 1. As can be seen, the results in this sample are rather good. Only the 5th and 6th questions were rated worse than the rest, which basically means that the version of the game used in the test was not well integrated and there were some inconsistencies. However, following evaluation the game was improved according to participants’ suggestions. The general score obtained by this questionnaire was 89.58 out of 100. This means that this game obtains an A grade, which is the best possible.

**Table 5:** SUS questionnaire results.

Question_ Number	jp176b	in143b	iu216b	co156b	lo163b	ai123b
Question 1	5	5	5	4	5	5
Question 2	1	1	1	1	1	1
Question 3	5	4	5	5	5	5
Question 4	1	1	1	1	1	1
Question 5	5	4	5	3	3	4
Question 6	1	2	1	3	3	2
Question 7	5	5	5	5	4	5
Question 8	1	1	1	1	1	1
Question 9	5	4	–	5	5	5
Question 10	1	2	–	1	1	1

## 6. Conclusions

This paper presents a game developed for iPad devices, whose aim of this therapy not only to improve the comprehension but also hold the attention of children with ADHD. Due to the fact that traditional methods of treating the symptoms of ADHD are not always effective, alternative methods are needed. Incorporation of Serious Games in therapy is one of them.

The review of literature and articles available on the Internet included in this paper shows that although there are many Serious Games aimed at helping children with ADHD, they usually focus on fostering attention. There are not many solutions which help in everyday life. Furthermore there are not many games and applications developed for iOS devices, especially the iPad, which are becoming more and more popular on the market. Therefore, it is hoped that the game presented in this report will fill this gap.

The results presented in Section 5 show that the game is user-friendly and easy to follow, which the score obtained from the SUS questionnaire confirms. The improved version meets all the requirements stated by participants in the evaluation. The main conclusions drawn from the study are:

(a) At the first stage of the game, participants explored how to play, and the rules were not very clear to them.
(b) Age and gender differences became less influential as the game developed.
(c) Age was significant only in the first game. This means that older participants completed the first game faster than younger ones, but that age was not influential in subsequent games.

Future lines for this study involve expanding the tool, which is currently being used in combination with other traditional techniques, to include more activities and introduce the element of randomness. Moreover, in terms of the results presented in the database, an analysis of progress should be included to illustrate how reading comprehension skills improved. Another thing which is indispensable for validating the game is evaluation of the tool on target-users-children with Attention Deficit Disorder.

In developing a novel and interactive tool, it is hoped that the game presented will help children with Attention Deficit Disorder improve their reading comprehension skills and, thus, improve school performance. Eventually, it is foreseen that the tool will improve the quality of life of those and their families.

## References

[1] Polanczyk G., de Lima M.S., Horta B.L., Biederman J., Rohde L.A. (2007). The worldwide prevalence of ADHD: A systematic review and metaregression analysis. Amer. J. Psychiat..

[2] Selkowitz M. (2009). ADHD.

[3] Farrar A. Attention-Deficit Hyperactivity Disorder–Popular Works. http://lernerbooks.com.

[4] Attention-Deficit/Hyperactivity Disorder (ADHD). Symptoms and Diagnosis. http://www.cdc.gov/ncbddd/adhd/diagnosis.html.

[5] American Psychiatric Association (2004). Diagnostic and Statistical Manual of Mental Disorders, Fourth Edition, Text Revision.

[6] (1993). The ICD-10 Classification of Mental and Behavioral Disorders.

[7] National Collaborating Centre for Mental Health (2009). The NICE Guideline on Diagnosis and Management of ADHD in Children, Young People and Adults–National Clinical Practice Guideline Number 72.

[8] Greenhill L.L., Posner K., Vaughan B.S., Kratochvil C.J. (2008). Attention deficit hyperactivity disorder in preschool children. Child Adolesc. Psychiatr. Clin. North Amer..

[9] McDonagh M.S., Peterson K., Thakurta S., Low A. (2011). Drug Class Review: Pharmacologic Treatments for Attention Deficit Hyperactivity Disorder.

[10] Ghelani K., Sidhu R., Jain U., Tannock R. (2004). Reading comprehension and reading related abilities in adolescents with reading disabilities and attention-deficit/hyperactivity disorder. Dyslexia.

[11] McInnes A., Humphries T., Hogg-Johnson S., Tannock R. (2003). Listening comprehension and working memory are imparied in attention-deficit hyperactivity disorder irrespective of language impairment. J. Abnorm. Child Psychol..

[12] Willcut E.G., Pennington B.F., Olson R.K., Chhabildas N., Hulslander J. (2005). Neuropsychological analyses of comorbidity between reading disability and attention deficit hyperactivity disorder: In search of the common deficit. Dev. Neuropsychol..

[13] Shankweiler D. (1999). Words to meanings. Sci. Stud. Read..

[14] Klingberg T., Forssberg H., Westerberg H. (2002). Training of Working Memory in Children with ADHD. J. Clin. Exp. Neuropsychol..

[15] Beck S.J., Hanson C.A., Puffenberger S.S., Benninger K.L., Benninger W.B. (2010). A Controlled Trial of Working Memory Training for Children and Adolescents with ADHD. J. Clin. Child Adolesc. Psychol..

[16] Prins P.J.M., Dovis S., Ponsioen A., Brink E.T., van der Oord S. (2011). Does computerized working memory training with game elements enhance motivation and training efficacy in children with ADHD?. Cyberpsychol. Behav. Soc. Netw..

[17] Shaw R., Lewis V. (2005). The impact of computer-mediated and traditional academic task presentation on the performance and behaviour of children with ADHD. J. Res. Spec. Educ. Needs.

[18] Kulman R. Generalization of game-based learning for children with adhd. Proceedings of The Annual American Psychological Association Convention.

[19] Programa de Entrenamiento de Instrucciones Escritas Nivel Medio, 2013. http://www.orientacionandujar.es/2013/05/23/programa-de-entrenamiento-de-intruccionesescritas-nivel-medio/.

[20] Ismail R., Jaafar A. Interactive scree-based design for dyslexic children. Proceedings of the International Conference on User Science and Engineering.

[21] De Urturi Z.S., Zorrilla A.M., Zapirain B.G. Serious game based on first aid education for individuals with Autism Spectrum Disorder (ASD) using android mobile devices. Proceedings of the 16th International Conference on Computer Games.

[22] Hourcade J.P., Natasha B.-R., Thomas H. (2012). Multitouch tablet applications and activities to enhance the social skills of children with autism spectrum disorders. J. Pers. Ubiquitous Comput..

[23] Saleh M.S., Aljaam J.M., Karime A., Elsaddik A. Learning games for children with intellectual challenges. Proceedings of the International Conference on Information Technology Based Higher Education and Training.

[24] El Khayat G.A., Mabrouk T.F., Elmaghraby A.S. Intelligent serious games system for children with learning disabilities. Proceedings of the 17th International Conference on Computer Games.

[25] Campigotto R., McEwen R., Epp C.D. (2013). Especially social: Exploring the use of an iOS application in special needs classrooms. Comput. Educ. Int. J..

[26] Lee S.C. Addressing children’s handwriting and visual perceptual difficulties using ipad applications in occupational therapy practice. Proceedings of the 7th International Convention on Rehabilitation Engineering and Assistive Technology.

[27] Cho B.H., Lee J.M., Ku J.H., Jang D.P., Kim J.S., Kim I.Y., Lee J.H., Kim S.I. Attention enhancement system using virtual reality and eeg biofeedback. Proceedings of the IEEE Virtual Reality.

[28] Amon K.L., Campbell A. (2008). Can children with AD/HD learn relaxation and breathing techniques through biofeedback video games?. Aust. J. Educ. Dev. Psychol..

[29] Bartolomé N.A., Zorrilla A.M., Zapirain B.G. A serious game to improve human relationships in patients with neuro-psychological disorders. Proceedings of the 2nd International IEEE Consumer Electronics Society’s Games Innovations Conference.

[30] Chuang T., Lee I., Chen W. Use of Digital Console Game for Children with Attention Deficit Hyperactivity Disorder. http://eric.ed.gov/?id=ED514772.

[31] Lim C.G., Lee T.S., Guan C., Fung D.S.S., Zhao Y., Teng S.S., Zhang H., Krishnan K.R. (2012). A brain-computer interface based attention training program for treating attention deficit hyperactivity disorder. PLoS ONE.

[32] Frutos-Pascual M., García Zapirain B., Méndez Zorrilla A. (2014). Adaptive tele- therapies based on serious games for health for people with time-management and organisational problems: Preliminary results. Int. J. Environ. Res. Public Health,.

[33] Ruiz-Manrique G., Tajima-Pozo K., Montañes-Rada F. Case Report: “ADHD Trainer”: The Mobile Application That Enhances Cognitive Skills in ADHD Patients. http://f1000research.com/articles/3-283/v1.

[34] Kirakowski J. The Use of Questionnaire Methods for Usability Assessment. http://sumi.ucc.ie/sumipapp.html.

[35] Davis F.D. (1989). Perceived usefulness, perceived ease of use and user acceptance of information technology. MIS Quart..

[36] Davis F.D., Venkatesh V. (2004). Toward preprototype user acceptance testing of new information systems: Implications for software project management. IEEE Trans. Eng. Manag..

[37] Brooke J. (1996). SUS—A quick and dirty usability scale. Usabil. Eval. Ind..

[38] Lin H.X., Choong Y.-Y., Salvendy G. (1997). A proposed index of usability: A method for comparing the relative usability of different software systems. Behav. Inform. Technol..

[39] Lund A.M. (2001). Measuring Usability with the USE Questionnaire. STC Usability SIG Newslett..

[40] Human Factors in Educational Software for Young Children. http://dare.ubvu.vu.nl/handle/1871/9782.

[41] Geneva Appraisal Questionnaire (GAQ): Format, Development, and Utilization. http://www.affective-sciences.org/system/files/webpage/GAQ_English_0.pdf.

[42] Henderson S., Yeow J. iPad in Education: A case study of iPad adoption and use in a primary school. Proceedings of the 45th Hawaii International Conference on System Sciences.

